# Selective removal of soluble FLT-1 using a high-affinity DNA aptamer for potential apheresis treatment of preeclampsia

**DOI:** 10.1038/s41598-026-50826-2

**Published:** 2026-04-27

**Authors:** Kensuke Owari, Haishun Piao, Miyuki Hori, Kazunobu Futami

**Affiliations:** TAGCyx Biotechnologies Inc, Komaba open laboratory 403, 4-6-1 Komaba, Meguro, Tokyo, 153-0041 Japan

**Keywords:** Biochemistry, Diseases, Drug discovery, Medical research

## Abstract

Preeclampsia (PE) is a pregnancy-specific hypertensive disorder that has been associated with elevated levels of soluble fms-like tyrosine kinase-1 (sFLT-1), which is thought to contribute to impaired angiogenesis by binding and neutralizing angiogenic factors such as Vascular Endothelial Growth Factor (VEGF) and Placental Growth Factor (PlGF). Selective removal of sFLT-1 from the maternal circulation is being explored as a potential therapeutic approach for PE. In this study, we developed TXB-0080, a high-affinity DNA aptamer containing two distinct artificial bases (Xenoligo platform), which binds sFLT-1 with an affinity of *K*_D_ = 0.084 ± 0.023 nM. TXB-0080 was immobilized onto Sepharose beads to construct an apheresis model that efficiently and specifically removed sFLT-1 from serum. The aptamer-conjugated beads exhibited stability against autoclaving and compatibility with clinical anticoagulants. Notably, it did not bind free PlGF but effectively captured sFLT-1 without interfering with its interaction with ligands. These findings support the feasibility of aptamer-based apheresis as a novel and targeted strategy to address sFLT-1–associated pathology in PE.

**Supplementary Information:**

The online version contains supplementary material available at 10.1038/s41598-026-50826-2.

## Introduction

Preeclampsia (PE) affects approximately 5% of pregnancies beyond 20 weeks of gestation and is characterized by hypertension and proteinuria, leading to maternal and fetal mortality^1,2^. Severe cases of PE can progress to HELLP syndrome (Hemolysis, Elevated Liver enzymes, and Low Platelets) and may result in complications such as seizures (eclampsia), cerebral hemorrhage, liver and kidney dysfunction, and hemolysis^2^. The incidence of PE is increasing, particularly among pregnant individuals of advanced maternal age^3^. Although symptomatic treatments, such as antihypertensive medications and anticonvulsants, exist, only planned early delivery currently serves as a definitive treatment for PE^4–6^.

Recent studies have identified soluble fms-like tyrosine kinase-1 (sFLT-1) as a contributing factor in the pathophysiology of PE^7–9^. While sFLT-1 levels gradually increase with gestational age in healthy pregnancies, its expression becomes markedly elevated in PE due to placental hypoplasia^10^. The median concentration of sFLT-1 in normal pregnancies is approximately 1000 pg/mL, whereas levels in PE patients are often significantly higher, commonly exceeding 8000 pg/mL (median) and in some cases reaching up to 50000 pg/mL^11,12^.

sFLT-1 is a soluble variant of vascular endothelial growth factor receptor 1 (VEGFR-1, also known as FLT-1), which lacks the transmembrane and intracellular domains due to ectodomain shedding or alternative splicing^13,14^. Although sFLT-1 is unable to transduce signals, it retains the ability to bind angiogenesis-inducing ligands such as Placental Growth Factor (PlGF) and Vascular Endothelial Growth Factor (VEGF) through its immunoglobulin (Ig) -like C2 type domains^15^. Consequently, excess sFLT-1 in circulation sequesters free PlGF and VEGF, inhibiting their biological activities^10^. This inhibition disrupts VEGFR signaling, contributing to vascular endothelial dysfunction and the clinical manifestations of PE^16^. In addition, in the kidneys, where continuous angiogenesis by VEGF is required, excessive VEGF binding by sFLT-1 is thought to cause kidney damage and proteinuria^17^.

Attempts to predict and diagnose the onset of PE have been made continuously. The use of the circulating sFLT-1/PlGF ratio as a diagnostic tool for the short-term prediction of PE is gaining recognition. The PROGNOSIS study demonstrated that, with a cut-off value of 38^18^, this ratio can predict the absence of PE within one week with a negative predictive value of 99.3%, and the occurrence of PE within four weeks with a positive predictive value of 36.7%. Additional studies have shown that this ratio is effective in detecting PE between 20 and 33 weeks of gestation (cut-off value of 85) and after 34 weeks (cut-off value of 110)^19^. These findings underscore the strong association between elevated sFLT-1 levels and the pathological conditions linked to PE, indicating that sFLT-1 may represent a potential therapeutic target in managing this condition.

Considering that sFLT-1 is a primary pathogenic factor in PE, several studies have focused on strategies to mitigate the adverse effects of elevated sFLT-1 levels, including the use of siRNA, low molecular weight compounds, and apheresis, alongside supplementing VEGF^20–25^. Clinical research has reported the use of dextran sulfate columns in apheresis to remove circulating sFLT-1, leveraging their electrostatic interaction charge^22^. However, this approach lacks specificity, as the column may also capture other positively charged components in the blood, potentially saturating the binding surface and reducing the overall efficiency of sFLT-1 removal. While anti-sFLT-1 antibodies could serve as specific ligands, challenges such as high production costs, lot-to-lot variability, and immunogenicity hinder their use as apheresis ligands. Consequently, there is a need for the development of optimal apheresis ligands that can specifically target sFLT-1.

DNA aptamers are single-stranded oligonucleotides that can form functional three-dimensional structures and bind to a wide range of targets, from metal ions to cell membrane proteins^26–29^. Due to their high specificity for targets, DNA aptamers have been applied in medical fields such as therapeutics and diagnostics, including apheresis ligands^30,31^. DNA aptamers are generated through a process called Systematic Evolution of Ligands by EXponential enrichment (SELEX) using a DNA library consisting of random sequences^32^. To enhance the functionality of DNA aptamers, Hirao et al. developed breakthrough technologies that incorporate the hydrophobic artificial base Ds (7-(2-thienyl)imidazo[4,5-b]pyridine), allowing for the amplification of Ds-containing DNA libraries by polymerase chain reaction (PCR) using another artificial base, Px (2-nitro-4-propynylpyrrole), which forms a base pair with Ds^33,34^. SELEX utilizing these Ds-containing DNA libraries has enabled the creation of high-affinity aptamers that exhibit *K*_D_ values in the picomolar range against protein targets^34,35^. The sequences of these aptamers can be optimized based on the results of secondary SELEX^34,36–38^. Additionally, the complete form of the aptamer was developed using Xenoligo technology by adding a mini-hairpin structure to the terminal end of the aptamer^36–38^. While unmodified DNA molecules are easily degraded by nucleases in blood^39^, the mini-hairpin structure provides nuclease tolerance to the aptamer by preventing exposure of the terminal regions, which is especially advantageous for systemic drugs or apheresis ligands that encounter blood during treatment^36,37^. In addition to these properties, the production of aptamers through Xenoligo chemical synthesis ensures no lot-to-lot variation and low immunogenicity, making it suitable for bulk production and application as apheresis ligands.

In this study, we conducted selection experiments using Xenoligo technology against sFLT-1 via the SELEX method and developed TXB-0080 through subsequent optimization. We also generated a TXB-0080-conjugated bead column and evaluated its ability to remove sFLT-1, as well as its specificity and other properties as an apheresis ligand for the development of PE treatment.

## Results

### SELEX targeting sFLT-1

SELEX-derived sequence information and the subsequent screening steps are summarized in Supplementary Tables S1–S3. Next-generation sequencing (NGS) of the ssDNA pools after the 7th round yielded approximately 1.6 million high-quality reads, which were grouped into 19 major sequence clusters based on sequence similarity. Clusters showing extremely biased or low-complexity base composition were excluded because such sequences are commonly enriched by PCR amplification rather than target binding.

From the remaining clusters, seven representative aptamer candidates with sufficient read counts and distinct predicted secondary structures were selected for initial functional screening. Their binding activities toward sFLT-1 were evaluated by ELOSA (Supplementary Fig. S1). Among them, flt-07, which possesses a stable stem structure enriched in G–C pairs (Supplementary Table S2), exhibited the highest sFLT-1-dependent signal and was therefore chosen for further optimization.

**Fig. 1:**
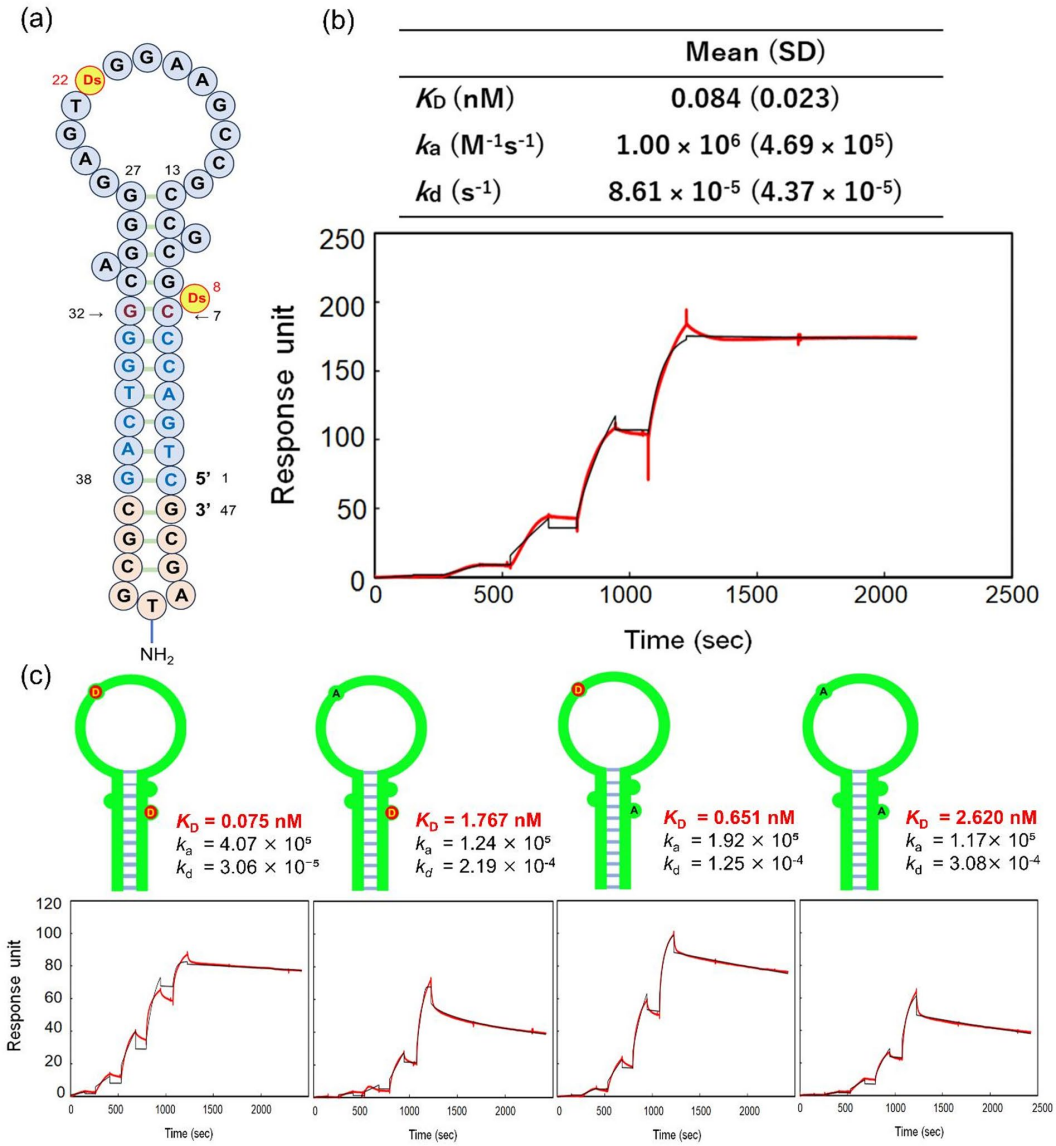
Structural and functional characterization of TXB-0080. **(a)** Schematic representation of the predicted secondary structure of TXB-0080. Highlighted in blue bases indicate the variable stem region; highlighted in red bases (positions 7 and 32) denote a fixed C–G pair; black bases are conserved. Nucleotide positions are numbered from the 5′ end. Black nucleotides in the loop region represent the core aptamer sequence selected during SELEX, and the same notation is used for the mini-hairpin sequence added after selection. Orange-shaded region indicates the introduced mini-hairpin structure. (**b**) Affinity measurement of TXB-0080 by SPR. TXB-0080 was immobilized on the sensor chip via an amino linker within the mini-hairpin. Shown are averaged sensorgrams from five independent measurements. Binding affinities were determined using a 1:1 Langmuir binding model (*n* = 5). (**c**) Contribution of Ds bases to the binding affinity of TXB-0080. Each Ds base was individually substituted with A, and the binding ability of each variant toward sFLT-1 was evaluated by *K*_D_ measurement. Data represents the mean of three independent experiments.

The final aptamer, TXB-0080, was obtained after 7 rounds of primary SELEX followed by 4 rounds of doped SELEX, and its affinity and structural stability were enhanced by appending a DNA mini-hairpin motif at the 3′ terminus. Its sequence and predicted secondary structure are shown in Fig. 1a. Two distinct artificial bases, Ds, were located within a stem and loop region potentially involved in target binding. Doped selection analysis demonstrated that the stem region of TXB-0080 was tolerant to sequence variation, provided that base pairing was maintained and the melting temperature remained within the optimal range. The C–G base pair at positions 7 and 32, however, was strictly conserved, with substitutions limited to a G–C pair. To facilitate subsequent analyses, an NH₂ linker was introduced at the mini-hairpin position.

### Characterization of anti-sFLT-1 DNA aptamer TXB-0080

The binding affinity of TXB-0080 to sFLT-1 was measured using Surface Plasmon Resonance (SPR). TXB-0080 was modified with a TEG linker-biotin via the mini-hairpin and covalently immobilized onto a streptavidin sensor chip. A 1:1 binding mode analysis revealed that TXB-0080 exhibited sub-nanomolar affinity (*K*_D_ = 0.084 ± 0.023 nM (*n* = 5), 1:1 Langmuir, global fit) (Fig. 1b).

To assess the contribution of the Ds bases in TXB-0080 to binding affinity, each Ds base was replaced with an A base, and the resulting variants were analyzed by SPR (Fig. 1c). The results demonstrated that the Ds base at position 8 was crucial for maintaining the structure, while the Ds base at position 22 was essential for preserving high affinity for sFLT-1, particularly by contributing to the low *k*_d_ value. Furthermore, the auxiliary sequence appended to the 3′ terminus to form a mini-hairpin structure did not affect the binding affinity or specificity of TXB-0080.

### Cross-reactivity with family proteins and ligand binding

**Fig. 2:**
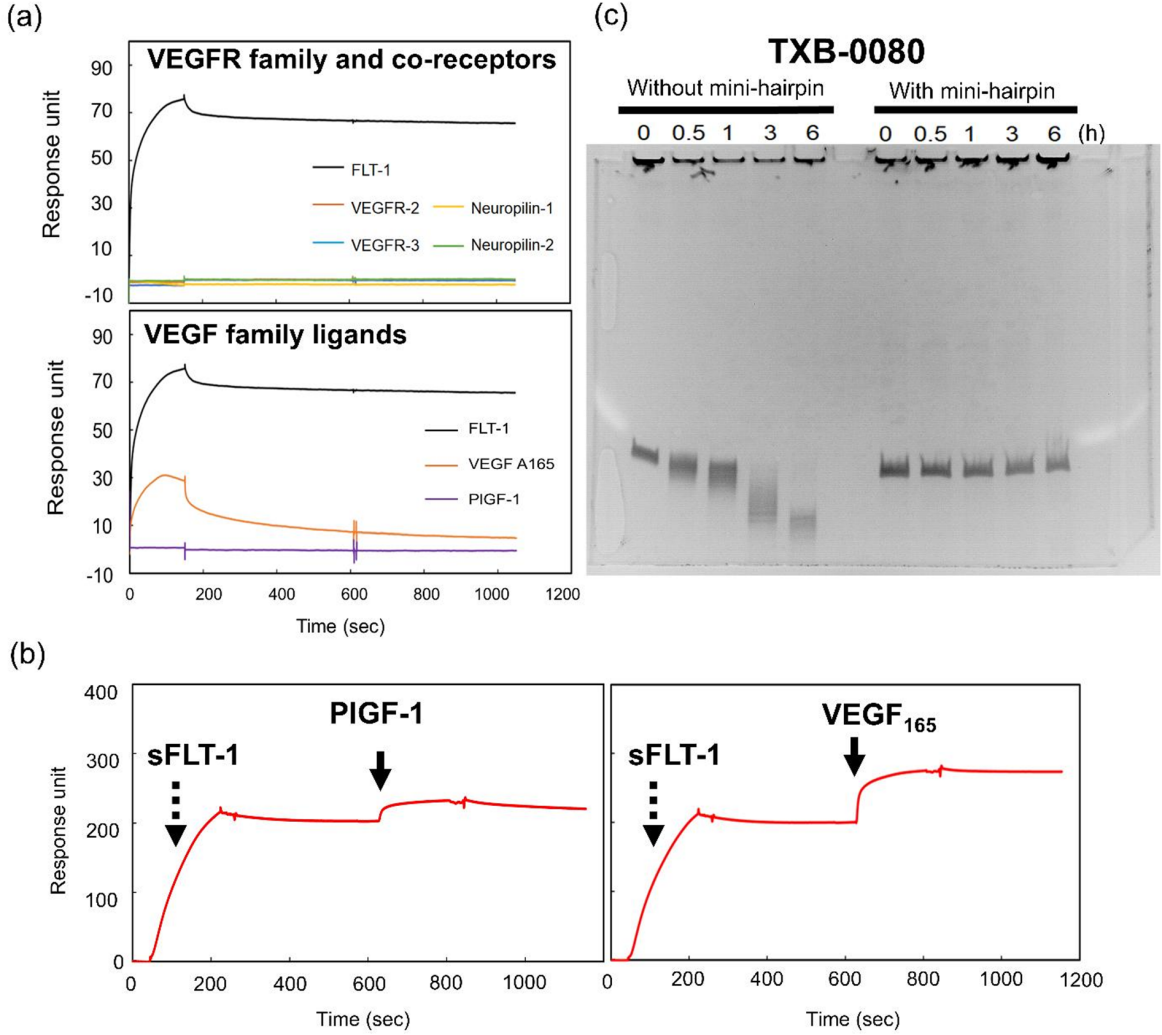
Specificity and stability of TXB-0080. (**a**) Specificity of TXB-0080 assessed by SPR. Sensorgrams were obtained using 100 nM of each VEGFR family protein and their co-receptors (upper panel), as well as 1000 nM of their respective ligands compared with sFLT-1 (lower panel). (**b**) Characterization of the FLT-1 binding domain recognized by TXB-0080. The binding of VEGF165 and PlGF-1 to FLT-1 was assessed by SPR. For sequential binding analysis, TXB-0080 was immobilized on the sensor chip, sFLT-1 was captured (dashed arrow), and VEGF165 or PlGF-1 was subsequently injected (arrows) to assess ligand binding. (**c**) Stability of TXB-0080 in human plasma. TXB-0080 with or without the mini-hairpin structure was incubated in human plasma, and aliquots were collected at the indicated time points. Samples were analyzed by denaturing PAGE followed by SYBR Gold staining. Figure 2c shows the full-length electrophoresis gel to ensure data integrity; original full-length images for all replicates are provided in the Supplementary Information file.

Next, we evaluated the specificity of TXB-0080 using other members of the VEGFR family, including VEGFR-2, VEGFR-3, Neuropilin-1, and Neuropilin-2, as analytes. At a concentration of 100 nM, none of these proteins, except for sFLT-1, exhibited measurable binding, indicating that TXB-0080 specifically binds to sFLT-1 among VEGFR family members (Fig. 2a). In addition, we examined the interaction of TXB-0080 with VEGF165 and PlGF isoform-1 (PlGF-1) at an excess concentration of 1000 nM. A weak binding signal was observed for VEGF165, whereas no detectable binding was observed for PlGF-1. This result suggests that the heparin-binding domain (HBD) of VEGF165 may interact with TXB-0080 through electrostatic interactions (Fig. 2a). Consistently, PlGF-1, which lacks the HBD, showed no binding, while PlGF isoform-2, which contains the HBD, exhibited detectable interaction (data not shown).

To determine whether TXB-0080 competes with ligand binding, SPR analyses were performed. Sequential binding was observed in that TXB-0080 first captured sFLT-1, and the subsequent addition of VEGF165 or PlGF-1 further increased the response units without dissociation of sFLT-1 from TXB-0080 (Fig. 2b). The differences in response magnitude and dissociation kinetics likely reflect the reported *K*_D_ values of VEGF (16 pM) and PlGF-1 (170 pM) for FLT-1^40,41^. These findings suggest that TXB-0080 does not compete with VEGF or PlGF-1 for their binding sites on FLT-1, and that it is unlikely to inhibit VEGFR-1 signaling in vivo, even if it enters systemic circulation. As previously mentioned, the concentration of free PlGF in circulation is reduced due to its capture by sFLT-1, and the resulting sFLT-1/PlGF imbalance contributes to the onset of PE by inhibiting VEGFR signaling necessary for placental angiogenesis. Therefore, it is crucial for PE treatment to avoid affecting PlGF levels.

Although direct quantification of sFLT-1–PlGF complexes was not feasible in this study, it is plausible that TXB-0080 may also recognize such complexes, in which PlGF is likely already unable to promote angiogenesis. Taken together, these findings demonstrate that TXB-0080 has significant potential for application in the treatment of PE through apheresis.

To further evaluate the molecular specificity of TXB-0080, cross-reactivity tests were performed with other human proteins. SPR analysis showed no significant binding of TXB-0080 to native human fibrinogen or recombinant human soluble Endoglin (sEng) (Supplementary Fig. S2). These results, together with the species specificity data, confirm that TXB-0080 highly specifically recognizes human sFLT-1 without non-specific interference from other major serum proteins or disease-related factors. Even at higher concentrations (1 µM), no significant binding was observed for native fibrinogen and sEng, contrasting with the high-affinity binding seen with sFLT-1 at much lower concentrations (*K*_D_ = 0.084 nM).

### Species Specificity

We next assessed the species specificity (cross-reactivity) among various species. As summarized in Supplemental Table S4, while primates exhibited a high degree of amino acid sequence identity, identity and homology both declined in more distantly related species. Binding affinities of TXB-0080 to the respective recombinant proteins were then evaluated. In crab-eating macaque (cynomolgus monkeys), although the amino acid sequence is highly homologous to humans, the binding affinity was slightly lower than that observed in humans, suggesting that the aptamer discriminates subtle structural differences derived from its target amino acid sequence (*K*_D_ = 0.423 ± 0.205 nM (*n* = 3), Fig. S3a). Although pigs (*K*_D_ = 10.2 ± 6.16 nM (*n* = 3)) and dogs (*K*_D_ = 28.9 ± 8.36 nM (*n* = 3)) showed reduced affinity, moderate binding was still observed (Fig. S3b, c). In rodents, the reactivity was minimal, and only weak, charge-related interactions were detected (Fig. S3d, e). These findings suggest that on-target toxicity studies of TXB-0080 using large animals are at least feasible.

### Serum stability

We evaluated the blood stability of TXB-0080 to assess its suitability for apheresis. The results showed that TXB-0080 remained stable in human plasma for up to 6 h, whereas a similarly sized sequence lacking the mini-hairpin structure degraded completely within the same period (Fig. 2c). Since apheresis treatments typically last around 2 to 3 h^42^, these results indicate that TXB-0080 possesses sufficient stability for the duration of the clinical procedure.

### sFLT-1 removal by TXB-0080-conjugated beads

For practical apheresis applications, we prepared TXB-0080–conjugated beads to capture sFLT-1 (Fig. S4). The estimated linker length between the bead surface and the aptamer was approximately 20 atoms (14 atoms from the adapter and 6 atoms from the C6 spacer). The conjugation reaction was monitored by qPCR (see Supplementary Methods), and approximately 10 pmol of TXB-0080 was immobilized per 1 mL of beads.

**Fig. 3:**
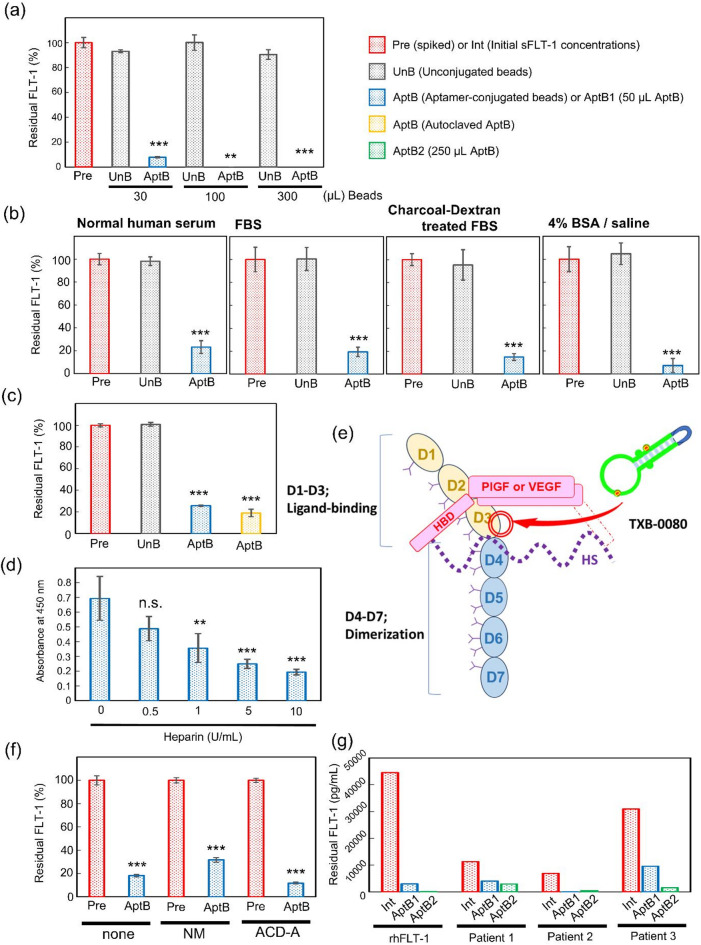
Efficient removal of sFLT-1 using TXB-0080-conjugated beads. (**a**) sFLT-1 (8000 pg in 1 mL FBS) was removed using 30, 100, or 300 µL of TXB-0080–conjugated beads. Equal volumes of unconjugated beads served as negative controls. Residual sFLT-1 levels quantified by ELISA are presented as mean ± SD (*n* = 3). ***p < 0.001, compared to the unconjugated beads group at each volume; Student’s t-test. (**b**) sFLT-1 (30000 pg recombinant human sFLT-1) in 1 mL of normal human serum, fetal bovine serum (FBS), charcoal–dextran-treated FBS, or 4% (w/v) BSA in saline was removed using 30 µL of beads. Residual sFLT-1 levels quantified by ELISA are shown as mean ± SD (*n* = 3). ***p < 0.001, compared to the unconjugated beads group at each media; Student’s t-test. (**c**) sFLT-1 removal activity of autoclaved or non-treated TXB-0080–conjugated beads or unconjugated beads was evaluated using 30 µL of beads and 30000 pg sFLT-1 in 1 mL FBS. Residual FLT-1 levels were quantified by ELISA and are presented as mean ± SD (*n* = 3–4). ***p < 0.001, compared to the unconjugated beads group; one-way ANOVA followed by Dunnett’s test. (**d**) Effect of heparin concentration on sFLT-1 capture using 30000 pg sFLT-1 in 1 mL FBS. Bound sFLT-1 on TXB-0080–conjugated beads were detected using an anti-His-tag HRP-conjugated antibody and HRP substrate. Data are presented as absorbance values at OD450 and shown as mean ± SD (*n* = 3). **p < 0.01, ***p < 0.001, n.s.: not significant, compared to 0 U/ml heparin group; one-way ANOVA followed by Dunnett’s test. (**e**) Schematic representation of the TXB-0080 binding site on FLT-1. The ligand-binding domains (D1–D3) and dimerization domains (D4–D7) are shown in yellow and blue, respectively. FLT-1 ligands (VEGF or PlGF, shown in pink) bind between domains D2 and D3, and their binding is promoted and stabilized by the heparin-binding domain (HBD) of each ligand. Naturally occurring heparan sulfate (HS, shown in purple) binds to the D3–D4 interface and is located near the TXB-0080 binding site. (**f**) Effect of other anticoagulants on sFLT-1 removal by TXB-0080–conjugated beads. NM; nafamostat mesylate, ACD-A; Acid Citrate Dextrose solution A. Residual sFLT-1 levels quantified by ELISA are presented as mean ± SD (*n* = 3). ***p < 0.001, compared to the Pre sample group at each anticoagulant; Student’s t-test. (**g**) Serum samples from three patients with preeclampsia (PE) patients were tested using TXB-0080–conjugated beads. The initial sFLT-1 concentrations (red bars) varied among samples (10000, 6000, and 30000 pg/mL). sFLT-1 levels after separation are shown as blue bars (50 µL beads) and green bars (250 µL beads). Abbreviations: Pre; spiked sFLT-1 before removal, UnB; Unconjugated beads, AptB; Aptamer-conjugated beads, Int; initial sFLT-1 concentrations, AptB1; 50 µL AptB, AptB2; 250 µL AptB.

In the separation experiment, recombinant sFLT-1 spiked into fetal bovine serum (FBS) at a concentration of 8000 pg/mL (the median concentration in the blood of patients with PE) was incubated with the TXB-0080-conjugated beads in a tube under gentle inversion (batch method). Different slurry volumes of beads (30, 100, and 300 µL) were tested, and even with 30 µL, approximately 92% of the spiked sFLT-1 was removed, leaving only 760 pg/mL detectable in the supernatant. Quantification of residual sFLT-1 in the supernatant by ELISA revealed that the TXB-0080-conjugated beads efficiently removed nearly all sFLT-1, whereas unconjugated beads exhibited minimal adsorption. Although the amount of TXB-0080 on 30 µL of slurry beads exceeded that of sFLT-1 (approximately 0.3 pmol of TXB-0080 versus 0.07 pmol of sFLT-1), complete depletion was not achieved, suggesting that not all immobilized TXB-0080 were functionally active, likely due to steric hindrance or surface density effects. These results confirm that TXB-0080 retained its binding capacity to sFLT-1, even when immobilized on a matrix (Fig. 3a).

To evaluate the effect of different dispersion media on sFLT-1 capture by TXB-0080, we increased the spiked sFLT-1 concentration to approximately 30000 pg/mL (about four times higher than in Fig. 3a) and tested binding using 30 µL of bead slurry—the minimum effective volume determined in Fig. 3a—under non-saturating conditions (Fig. 3b). TXB-0080–conjugated beads efficiently captured sFLT-1 in all media tested, whereas unconjugated beads captured only trace amounts. However, a slight reduction in capture efficiency was observed in native biological matrices such as human serum and FBS compared with the minimal composition of 4% BSA in physiological saline (capture efficiencies; 77% in human serum, 81% in FBS, 85% in Charcoal-dextran treated FBS and 95% in 4%BSA/saline). This reduction is likely attributable to interference from charged components present in the complex matrices.

### Applicability of TXB-0080-conjugated beads in Apheresis

To evaluate the suitability of the TXB-0080 conjugated separation beads for apheresis applications, additional studies were conducted. Currently, gamma-irradiation and autoclaving are becoming the mainstream sterilization methods for apheresis devices, replacing ethylene oxide gas. Since Sepharose beads are autoclavable according to the supplier’s manual, and DNA aptamers exhibit thermal stability, we investigated the applicability of autoclave sterilization to the TXB-0080 beads. TXB-0080-conjugated beads were autoclaved in 1x D-PBS and subsequently, after which the separation capacity of the beads was assessed by repeating the FBS-based separation experiment in batch method shown in Fig. 3b. As anticipated, autoclaving did not impair its separation capacity, indicating that TXB-0080 maintained its structural integrity post-autoclaving. Therefore, autoclave sterilization is suitable for actual devices utilizing TXB-0080 (Fig. 3c).

Next, we investigated the effects of various additives on the properties of the TXB-0080-conjugated beads, particularly anticoagulants. During apheresis, anticoagulants such as heparin and Acid Citrate Dextrose solution-A (ACD-A) are utilized to separate plasma and prevent blood clotting throughout the treatment^43^. In addition to these anticoagulants, we also examined a general anticoagulant, and nafamostat mesylate (NM), a synthetic serine protease inhibitor frequently used as an anticoagulant in Japan^44^. The removal capacity of recombinant sFLT-1 was assessed in the presence of these anticoagulants at clinically relevant doses.

(1) Heparin.

FLT-1, the binding target of TXB-0080, is a membrane-associated receptor that interacts with extracellular matrix components. Domains 3–4 of FLT-1 contain a heparan sulfate–binding site^45^, structurally similar to heparin, which mediates interactions with ligands such as VEGF and PlGF. Moreover, the extracellular domain of FLT-1 is overall positively charged, raising the possibility that heparin, when administered as an anticoagulant, may coat the receptor through electrostatic interactions and thereby interfere with TXB-0080 binding. To examine this hypothesis, various concentrations of heparin (normally 0.4–1.5 U/mL- blood in apheresis procedure), including those exceeding physiological levels, were added during the capture of spiked sFLT-1 by TXB-0080-conjugated beads. Because higher concentrations of heparin interfered with antibody-based ELISA detection of sFLT-1 (data not shown), binding was assessed by ELOSA under conditions that minimized such interference. As shown in Fig. 3d, TXB-0080 binding began to show measurable inhibition at 0.5–1 U/mL heparin. Nevertheless, even at 10 U/mL, more than ~ 25–30% of the capture activity was retained, indicating that the interference was partial rather than complete. These findings suggest that the recognition site of TXB-0080 at least partially overlaps with the heparin-binding domains of FLT-1.

Taken together, these results suggest that the TXB-0080 binding region partially overlaps with the heparin-binding site, but does not overlap with the ligand-binding domain (Fig. 2b). Given the nature of aptamers, it is likely that TXB-0080 binds to a non-surface region—specifically, a crevice within Ig-like C2 type 3—avoiding the glycosylation site located on the backside (see Fig. 3e). The artificial base Ds is presumed to engage in π–π stacking interactions within hydrophobic pockets or grooves of the protein, which contain aromatic residues; however, further detailed analyses are needed.

These findings suggest that the use of heparin as anticoagulant may partially interfere with aptamer binding, thereby reducing the efficiency of sFLT-1 removal.

(2) Other anti-coagulation reagents.

The final concentrations of NM (0.02 mg/ml) were calculated based on the assumption that 50 mg/hour of NM (maximum dose) in 2 h treatment, administered continuously per hour during apheresis treatment, were homogeneously distributed in an average adult blood volume of 4.5 L. ACD-A was set to concentrations recommended in clinical practice. Of the anticoagulants tested, NM slightly affected the sFLT-1 removal capacity of TXB-0080 (Fig. 3f). Additionally, NM contains a naphthalene ring that may interact with DNA bases^46,47^, suggesting that it could potentially affect the structure of TXB-0080. Although these anticoagulants impacted removal efficiency in single-pass experiments, the removal of sFLT-1 can still be achieved through multiple cycles of extracorporeal circulation.

### Effectiveness of TXB-0080 in Removing Endogenous sFLT-1

Although previous experiments used recombinant sFLT-1 to mimic circulating sFLT-1 in PE, we further examined whether the TXB-0080-conjugated beads could effectively remove endogenous sFLT-1 derived from the serum of PE patients. To model a patient with sFLT-1 levels of 50000 pg/mL, an equivalent amount of recombinant sFLT-1 was spiked into FBS and used as a control for apheresis experiments. The TXB-0080-conjugated beads were tested at two different slurry volumes, 50 or 250 µL against 250 µL serum. Serum from three commercially available PE patients was used for the study.

The baseline sFLT-1 levels in the PE patient sera were 10000, 6000, and 30000 pg/mL, respectively. In the control experiments with FBS spiked with 50000 pg/mL of recombinant sFLT-1, efficient removal was achieved even with 50 µL of beads (93% capture). However, the removal efficiency varied among the PE patient sera. In patient 1, only 64% of sFLT-1 was removed, and even after increasing the bead volume fivefold, 24% of sFLT-1 remained. In contrast, efficient removal was achieved for patient 2 (94% capture in 50 µL beads and below detectable limit in 250 µL beads). For patient 3, who had a high baseline sFLT-1 level, using 50 µL of beads left 10000 pg/mL of sFLT-1, indicating that additional bead volume would be required to reduce sFLT-1 to the normal range (Fig. 3g). These results suggest that factors inherent to PE patient sera, rather than heparin, may hinder sFLT-1 removal. Nevertheless, this limitation could potentially be overcome by increasing the concentration of TXB-0080-conjugated beads or by performing repeated removal cycles.

These findings collectively indicate that TXB-0080 specifically targets sFLT-1, supporting its potential utility in therapeutic apheresis for PE.

## Discussion

In this study, we developed and validated TXB-0080, a high-affinity DNA aptamer targeting human sFLT-1, and demonstrated its utility for specific removal of sFLT-1 using a Sepharose-based apheresis column.　In vitro studies have demonstrated the validity of this concept, showing that TXB-0080 has been successfully utilized in apheresis applications to remove sFLT-1 from serum. The efficacy of apheresis for eliminating excess sFLT-1 in plasma has already been established, with undeniable benefits for pregnant women. However, none of these applications have reached the commercial level due to several challenges associated with the medical use of apheresis columns containing DNA aptamers.

One major challenge is the strict species specificity of DNA aptamers, which complicates validation in animal models. Verification in animal models to observe improvements in hypertensive nephropathy has yet to be achieved. Additionally, there are concerns regarding the suitability of Sepharose beads used in this study as immobilization resin for clinical specimens.

Despite these challenges, the application of DNA aptamers in apheresis columns presents several advantages that may help overcome these obstacles. The benefits of DNA aptamers include: (1) the ability to be chemically synthesized for specific applications, (2) low antigenicity, (3) non-transferability from mother to fetus, (4) excellent storage stability across a range of temperatures, and (5) compatibility with autoclave sterilization. Notably, the exceptionally high association rate (*k*a = 1.00 × 10^6^ M^−1^s^− 1^) and high affinity (*K*_D_ = 0.084 nM) of TXB-0080 suggest its capacity for efficient sFLT-1 capture even under the rapid flow conditions required for clinical apheresis, where residence time within the column is limited. In clinical settings, the clearance efficiency can be further managed by optimizing the column geometry and bed volume relative to the patient’s blood flow rate. Furthermore, although TXB-0080 binds to both free and ligand-complexed sFLT-1, our therapeutic strategy focuses on the rapid and significant reduction of the total sFLT-1 burden. In severe preeclampsia, the pathological excess of sFLT-1 is so overwhelming that a drastic reduction in the total pool is necessary to effectively restore the systemic angiogenic balance. By rapidly lowering the total sFLT-1 concentration, we facilitate the restoration of free VEGF and PlGF levels from endogenous sources, a process ensured by the high binding performance of TXB-0080 compared to conventional non-specific sorbents, such as dextran sulfate columns.

Compared with antibody-based capture systems, aptamer-functionalized columns offer several advantages, including thermal stability, cost-effectiveness, and ease of chemical modification. Given the high specificity of TXB-0080 and its resistance to common anticoagulants, this system may be particularly useful in clinical settings where minimal off-target adsorption is critical. Regarding the clinical safety of the column, the high stability of TXB-0080 shown in Fig. 2c suggests that significant aptamer leakage is unlikely during the typical 2- to 3-hour duration of apheresis procedures^42^. Importantly, even if trace amounts of the aptamer were to leak or undergo degradation, the resulting nucleotides or short oligomers are endogenous substances that are rapidly cleared by renal excretion, posing minimal risk of systemic toxicity. This represents a significant safety advantage of aptamer-based columns over synthetic polymer-based systems.

However, we acknowledge that our clinical validation was limited to a small cohort (*n* = 3), and the clinical characteristics of these patients, including gestational diabetes mellitus (GDM) in all cases, are summarized in Supplementary Table S5. In PE patients, particularly those with comorbid GDM, complex serum biochemical profiles—characterized by elevated inflammatory cytokines, altered lipid metabolism, and increased oxidative stress—could potentially interfere with aptamer-sFLT-1 interactions. While TXB-0080 demonstrated consistent performance in this study, future investigations with a larger and more diverse population are essential to confirm the robustness of this approach across various clinical presentations.

Furthermore, it should be noted that apheresis is an invasive procedure requiring specialized equipment and trained personnel. Therefore, while this technology offers a promising therapeutic option, its application may currently be limited to specialized tertiary centers and may face implementation challenges in low- and middle-income healthcare settings.

In conclusion, the concept of integrating the advantages of aptamers with column chromatography for the development of specific sorbents is highly promising. Despite the current clinical and technical challenges, TXB-0080-based apheresis columns represent a significant step toward a more specific and stable treatment for preeclampsia.

## Materials and methods

### SELEX

Described in Supplementary information.

### Aptamer Synthesis

The final lead aptamer, TXB-0080 (CTGACCCDsGCGCCGCCGAAGGDsTGAGGGGACGGGTCAGCGCGT^*^AGCG), was synthesized by solid-phase oligonucleotide synthesis and purified by HPLC (GeneDesign, Osaka, Japan). An amino-modified linker or a biotin moiety was directly introduced at the position indicated by “T^*^” during synthesis.

### SPR measurements

All binding analysis were conducted using Biacore T200 (Cytiva, MN, USA) in 1x D-PBS supplemented with 0.05% (wt/vol) Tween 20 and additional 100 mM NaCl (final concentration of 237 mM) to prevent nonspecific binding to Biacore sensor surface caused by strong positive charge of FLT-1. The measurement temperature was set at 25℃. To confirm affinity of selected aptamer (TXB-0080), biotinylated TXB-0080 via mini-harpin was immobilized on a Sensor Chip SA (Cytiva, MN, USA), and the interaction of the immobilized DNA aptamer with recombinant human FLT-1 was detected by monitoring injections of 0.01, 0.39, 1.56, 6.25 and 25 nM FLT-1 (Sino Biological, Beijing, China) solutions (diluted with the running buffer) using single-cycle kinetics. The conditions were flow rate: 100 µl /min, protein injection time: 150 s, and dissociation time: 900 s. The data was analyzed with a 1:1 kinetic binding model using the BIAevaluation T200 software, version 3.0 (Cytiva, MN, USA) to determine binding affinity (*K*_D_). The red sensorgrams represent the raw data, and the black curves show the fitted data based on a 1:1 binding model.

To determine Ds base contribution, each biotin modified variant sequence was immobilized SA sensor chip. The binding kinetics between TXB-0080 and sFLT-1 were measured using serial dilutions of sFLT-1 (0.39, 1.56, 6.25, 25, and 100 nM). The dissociation time was extended to 1200 s to accurately determine the dissociation rate constant (*k*_d_). To evaluate the molecular specificity of TXB-0080, 100 nM recombinant human VEGFR-2, VEGFR-3 (Sino Biological, Beijing, China), Neuropilin-1, and Neuropilin-2 (R&D Systems, MN, USA), or 1000 nM (1 µM) VEGF165 (PeproTech, NJ, USA), PlGF (R&D Systems, MN, USA), native human fibrinogen (Sigma-Aldrich, St. Louis, MO, USA), and soluble Endoglin (sEng; Sino Biological, Beijing, China) prepared in running buffer were injected. For epitope mapping, 100 nM recombinant sFLT-1 was captured via biotinylated TXB-0080 on a Sensor Chip SA (~ 200 RU immobilized). After washing, 50 nM VEGF165 or PlGF was loaded to assess competitive binding.

To evaluate species specificity, FLT-1 proteins from Crab-eating macaque (Thermo Fisher Scientific, CA, USA), rat and mouse (Sino Biological, Beijing, China), and pig and dog were examined. For Crab-eating macaque, pig and dog, cDNA sequences corresponding to the registered homologous FLT-1 genes (identified by sequence alignment) were synthesized and expressed in mammalian cells, since commercial recombinant proteins were not available. The *K*_D_ values were measured as described above. The accession numbers of each protein are listed in Supplementary Table S4.

### Stability analysis of aptamer in human plasma

TXB-0080 (final concentration: 720 nM, the aptamers were initially dissolved in 1x D- PBS) was incubated with human Heparin-Na plasma (Biopredic International, Saint Grégoire, FR) in 96% (v/v) concentration at 37 °C. Aliquots (20 µL) of reaction solution were removed at various time points from 0 to 6 h, and degradation was terminated by immediate mixing with 110 µl of denaturing solution (1×TBE containing 10 M urea). Each sample was fractionated by 12% denaturing polyacrylamide gel electrophoresis. The DNA was stained with SYBR Gold (Thermo Fisher Scientific, CA, USA). Full-length electrophoresis gel images, including the gel edges, are provided in the Supplementary Information file to ensure data integrity.

### Removal study of FLT-1 by TXB-0080 immobilized beads

To confirm aptamer function on a matrix, 30, 100 or 300 µL TXB-0080 immobilized beads (Supplementary Fig. S4) or unconjugated control beads were mixed with 1 ml Fetal Bovine Serum (FBS) spiked with 8000 pg recombinant human sFLT-1 (Sino Biological, Beijing, China) with gentle inversion at room temperature for 30 min. To evaluate dispersion media effect, 30 µL TXB-0080 beads or unconjugated beads were analyzed with 1 ml of 30000 pg recombinant human sFLT-1 spiked-FBS, Normal Human serum (Merck Millipore, MA, USA), Charcoal/Dextran Treated FBS (Cytiva, MN, USA) or 4% (w/v) Probumin (Merck Millipore, MA, USA) diluted in Otsuka Normal Saline (Otsuka Pharmaceutical Factory, Tokushima, Japan). For thermal stability evaluation, TXB-0080-conjugated beads were suspended in 1x D-PBS and autoclaved at 121℃ for 20 min. To confirm the effect of anticoagulant on sFLT-1 separation, 0–10 U/mL Heparin-Na (Mochida Pharmaceutical, Tokyo, Japan), 11% (v/v) ACD (Acid Citrate Dextrose)-A solution (2.20% (w/v) trisodium citrate dihydrate, 0.8% (w/v) citric acid monohydrate, 2.20% (w/v) glucose, in-house prepared) and 0.02 mg/mL Nafamostat Mesylate (FUTHAN) (Nichi-Iko Pharmaceutical, Toyama, Japan) were added to 1 mL FBS spiked with 30000 pg recombinant human sFLT-1. In the study using PE serum, 250 µL serum (ProteoGenex, CA, USA) was mixed with 50 or 250 µL beads. In all experiments except the confirmation of heparin effect, sFLT-1 removal efficiencies were measured by the sFLT-1 concentration in the supernatant by ELISA (R&D Systems, MN, USA). In the heparin effect study, sFLT-1 bound beads were directly colored by His-Tag Horseradish Peroxidase-conjugated Antibody and HRP substrate (R&D Systems, MN, USA) and OD_450_ of colored solution was measured.

### Human Serum Samples

Commercially available non-pregnant normal human serum samples were purchased from Merck Millipore (Burlington, MA, USA; Cat. No. S1-100ML), and PE patient serum samples were purchased from ProteoGenex (Los Angeles, CA, USA). All samples were collected under protocols approved by the Institutional Review Board (IRB) of the respective suppliers, and written informed consent was obtained from all donors prior to sample collection. All samples were fully anonymized by the suppliers before delivery. The PE serum samples were collected at different gestational stages: 29 weeks (Patient 1), 32 weeks (Patient 2), and 36 weeks (Patient 3).

### ELISA

The amount of protein remaining in serum was quantified using ELISA. On the sFLT-1; Human VEGFR-1/FLT-1 Duoset ELISA development system (R&D systems, DY321B) was employed.

### Statistical analysis

All the data are presented as the means ± standard deviations (SD) except Fig. 3g (*n* = 1). Statistical analysis was performed using Microsoft Excel (Microsoft Corporation, Redmond, WA, USA) or EZR^48^. F test or Bartlett’s test was carried out to assess the equality of variance in different groups. Differences with P-values < 0.05 were considered statistically significant using Student’s t-test (two-tailed) or one-way ANOVA followed by Dunnett’s test for multiple comparisons.

## Supplementary Information

Below is the link to the electronic supplementary material.

Supplementary Material 1

## Data Availability

The raw SELEX sequencing data generated in this study have been deposited in the NCBI Sequence Read Archive (SRA) under BioProject accession number PRJNA1398950 (www.ncbi.nlm.nih.gov) and SRA accession number SRR36882081.
