# Supplementary material for: Selective removal of soluble FLT-1 using a high-affinity DNA aptamer for potential apheresis treatment of preeclampsia
Source: Sci Rep. 2026 Apr 27;16:19434. doi: 10.1038/s41598-026-50826-2 (PMC13287809; doi:10.1038/s41598-026-50826-2)
Supplement: Supplementary file 1 — Supplementary Material 1 [file 41598_2026_50826_MOESM1_ESM.pdf]

Supplementary information

Selective removal of soluble FLT-1 using a high-affinity DNA aptamer for potential apheresis treatment of preeclampsia

Kensuke Owari, Haishun Piao, Miyuki Hori, and Kazunobu Futami*

TAGCyx Biotechnologies Inc., Komaba open laboratory 403, 4-6-1 Komaba, Meguro, Tokyo 153-0041, Japan.

* Correspondence should be addressed to K.F. (kfutami@tagcyx.com)

Table of Contents

Supplementary Tables S2

Supplementary Figures S7

Supplementary Methods S11

Original images for electrophoresis gels S14

Supplementary Table S1. SELEX Summary

Round	Target Used	Negative Selection	Wash
1	FLT-1-Fc / Protein G	human IgG / Protein G	1x D-PBS-T x 5 times, 4°C
2	FLT-1-Fc / Protein G	human IgG / Protein G	2x conc. D-PBS-T x 5 times, RT
3	FLT-1-Fc / Protein G	human IgG / Protein G	2x conc. D-PBS-T x 5 times, RT
4	FLT-1-His / TALON	TALON	2x conc. D-PBS-T x 5 times, RT
5	FLT-1-His / TALON	TALON	2x conc. D-PBS-T x 5 times, RT
6	FLT-1-His / TALON	TALON	2x conc. D-PBS-T x 5 times, RT
7	FLT-1-Fc /Protein G	human IgG / Protein G	2x conc. D-PBS-T x 5 times, RT

RT; Room temperature

Supplementary Table S2. 7 Candidate sequences selected from SELEX

Clone ID.	Random sequence (5' -> 3', common primer sequence at both ends)
flt-04	---CCCGCGAGGTTTGCTTAGGTCTGCTAGGAXGGTAGT---
flt-05	---CCCGGXTGAAAGCGCCGCCAACGGXTGAGGGGTCTT---
flt-07	---CCCCCXGCGCCGCCGAAGGXTGAGGGGACGGGGGAG---
flt-08	---CCCAAXTCGGACAGGTATGXTAGGACTGCTTAGGCT---
flt-09	---CCCGGXTAGGGCTTGACGGXCAGTCAGCGTGTCCTA---
flt-10	---AGTTCTAATACCCXTTTGAGGATTGCXCAGGTCTGC---
flt-11	---CCGCTTAAXGATTGTTAXGTTGTACGAAACGCATTC---

X; dDs

Supplementary Table S3. Secondary (Doped) SELEX Summary

Round	Target Used	Negative Selection	Wash
1	FLT-1-His / TALON	TALON	2x conc. D-PBS-T x 5 times, RT
2	FLT-1-His / TALON	TALON	2x conc. D-PBS-T x 5 times, RT
3	FLT-1-His / TALON	TALON	2x conc. D-PBS-T x 5 times, RT
4	FLT-1-His / TALON	TALON	2x conc. D-PBS-T x 5 times, RT

Supplementary Table S4. Comparison of VEGFR1 ECD amino acid sequences across species.

Organism	Refseq	Amino asids	Extracellular domain (position)	Percentage identity (%)
Homo sapiens<br>(Human)	NP_002010	1338	27 - 758	--
Macaca fascicularis<br>(Crab-eating macaque)	XP_005585612	1338	27 - 758	97.0%
Sus scrofa<br>(Pig)	XP_001925775	1337	27 - 756	83.9%
Canis lupus familiaris<br>(dog)	XP_038290894	1337	27 - 758	87.0%
Rattus norvegicus<br>(Rat)	NP_062179	1336	23 - 758	78.3%
Mus musculus<br>(Mouse)	NP_034358	1333	23 - 759	77.6%

Sequence identity and homology percentages were calculated based on alignment with the human VEGFR1 reference. For species other than human, rat and mouse, the domain 3 sequences were inferred from predicted protein models based on sequence alignment.

Supplementary Table S5. Baseline characteristics and clinical history of preeclampsia patients.

**Parameter**	**Patient 1**	**Patient 2**	**Patient 3**
Age (years)	36	28	37
Ethnicity	Caucasian	Caucasian	Caucasian
Trimester	3rd trimester	3rd trimester	3rd trimester
Weeks of gestation	29	36	32
**Baseline serum sFLT-1 (pg/mL)**	**11258**	**6874**	**30956**
Complications during pregnancy	Gestational diabetes mellitus, angiitis	Gestational diabetes mellitus, vegetative-vascular dystonia (hypotonic type), angiitis	Gestational diabetes mellitus
Current medications	Iodomarin, Aquadetrim,	Eutirox, Canephron, Clexane	Insulin, Eutirox, Norvax
Medical history	Hereditary thrombophilia	Hypothyroidism	Hypertension, hypothyroidism
Smoking status	No	No	No

The clinical profiles of the three patients (n = 3) used for the sFLT-1 removal experiments are presented. Baseline serum sFLT-1 concentrations were measured prior to the apheresis model experiments. Abbreviations: PE, preeclampsia; sFLT-1, soluble fms-like tyrosine kinase-1.

Note: Medication names are listed by their brand names as recorded in the clinical charts.

(a)

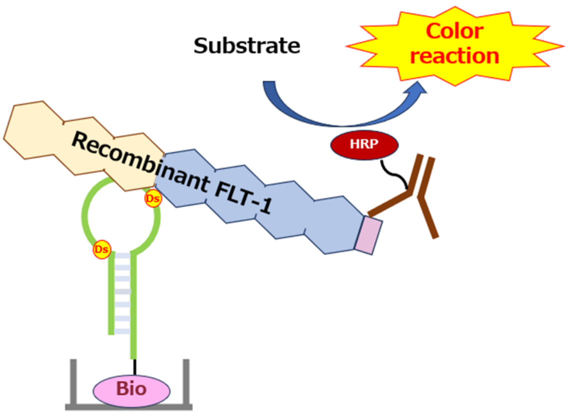

(b)

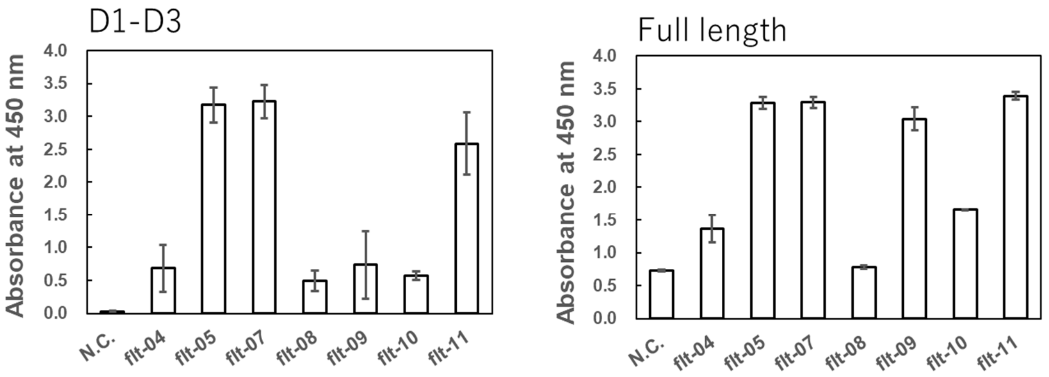

Supplementary Figure S1. Binding analysis of aptamer candidates by ELOSA.

(a) Schematic overview of the ELOSA system. Biotinylated aptamers were immobilized on NeutrAvidin-coated plates and incubated with recombinant FLT-1 proteins to allow capture. The binding event was subsequently detected via colorimetric signal.

(b) Comparison of binding signals of 7 candidate aptamers (flt-05, -07, -09, etc.). OD450 values represent the mean ± SD from triplicate measurements. Binding was assessed against two FLT-1 constructs: (left) the D1–D3 domain and (right) the full extracellular domain (Full FLT-1; D1-D7). TXB-0080 was not assessed at this stage. Background absorbance from wells without immobilized FLT-1 was subtracted as a blank. A random sequence aptamer was used as a negative control.

(a) Native human fibrinogen

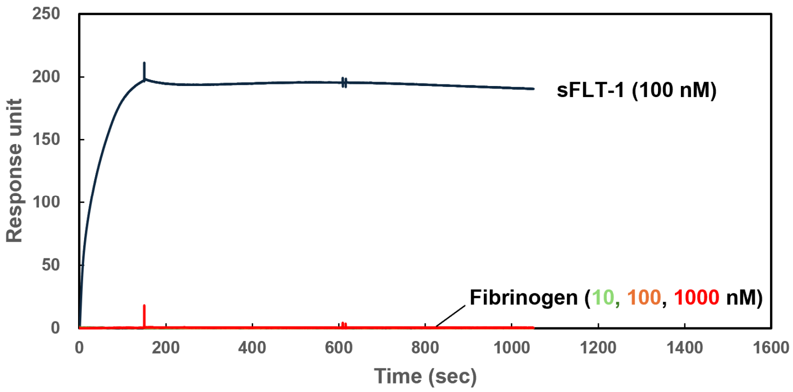

(b) Soluble Endoglin

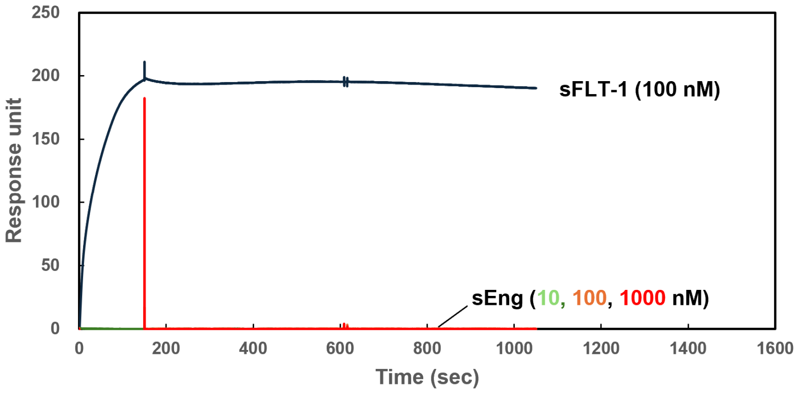

Supplementary Figure S2. Specificity and cross-reactivity analysis of TXB-0080.

SPR sensorgrams evaluating the cross-reactivity of TXB-0080. (a) Representative SPR sensorgram for native human fibrinogen. (b) Representative SPR sensorgram for recombinant human soluble Endoglin (sEng). Even at a high concentration of 1 μM, TXB-0080 showed no significant binding to these proteins, contrasting with the high-affinity binding observed with human sFLT-1 at much lower concentrations. These results demonstrate the high molecular specificity of TXB-0080, relying on shape-specific recognition rather than non-specific electrostatic interactions.

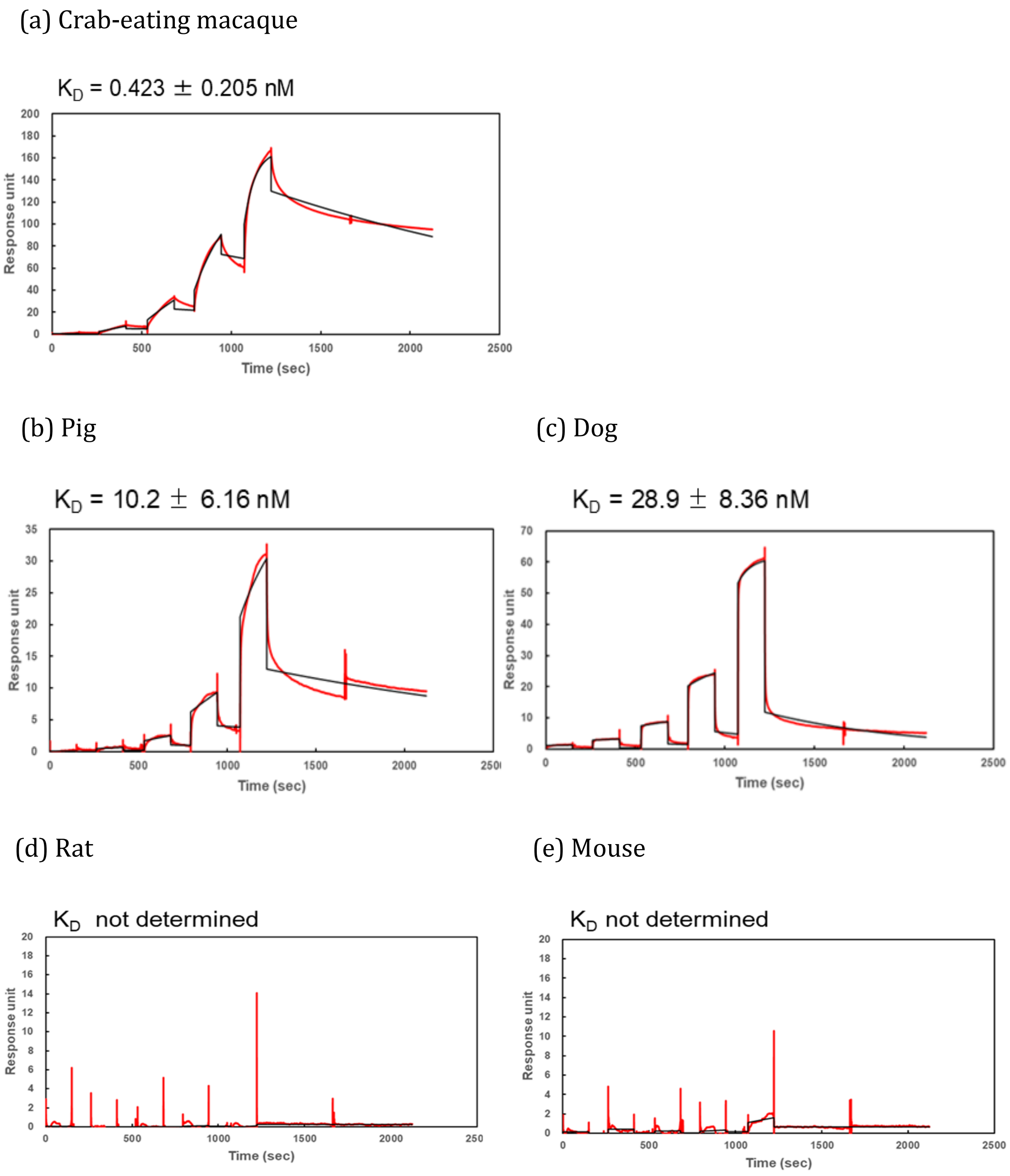

Supplementary Figure S3. Affinity analysis of TXB-0080 against FLT1 orthologs.

Recombinant soluble FLT1 extracellular domains from five non-human species were injected at various concentrations onto an aptamer-immobilized SA chip. Sensorgrams were globally fitted to a 1:1 binding model. Dissociation constants (K_D) are presented as mean ± SD from three independent experiments (n = 3). Representative sensorgrams are shown.

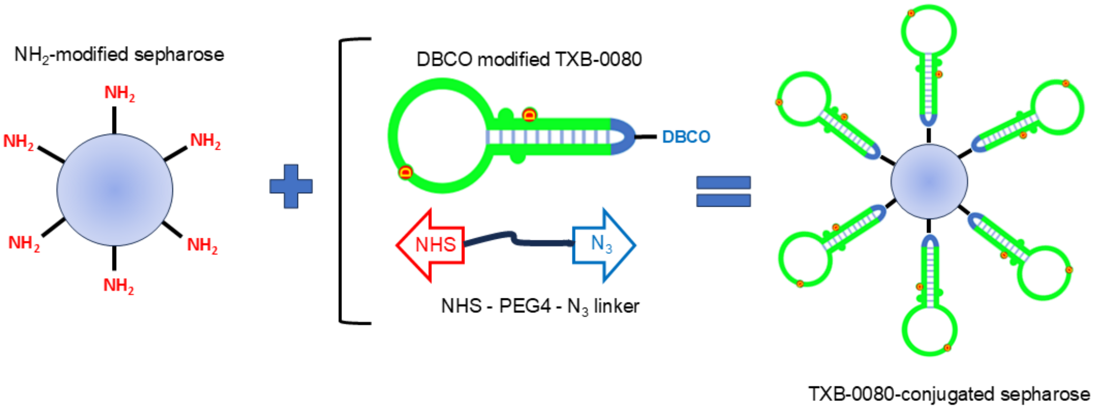

Supplementary Figure S4. TXB-0080 conjugated Sepharose preparation.

TXB-0080 was first modified with a DBCO group at the amino linker introduced at the 3′ mini-hairpin end. The resulting TXB-0080-DBCO was then covalently attached to amino-modified Sepharose beads via an azide-PEG4-NHS adapter, forming a stable triazole linkage through strain-promoted azide-alkyne cycloaddition (SPAAC).

Supplementary Methods：

SELEX for sFLT-1 Specific Aptamer (TXB-0080)

Library Construction

A partially randomized single-stranded DNA library containing two defined artificial hydrophobic bases (Ds: 7-(2-thienyl)imidazo[4,5-b]pyridine) was synthesized, following the method described by Futami et al. The randomized region (36 nt) contained two predefined Ds positions. The library was flanked by constant primer regions to enable PCR amplification using Px/Ds compatible bases.

Target Immobilization and Negative Selection

Recombinant human FLT-1 proteins with His-tag (Sino Biological, Beijing, China, #10136-H08H1, (Ser27 – Ile328) - His) and Fc-tag (R&D systems, MN, USA #3516-FL-050, (Ser27 – Ile328) - Fc) were immobilized on TALON (Takara Bio, Shiga, Japan, #635501) or Protein G Sepharose beads (Protein Ark, Rotherham, UK. #Fastback-PG-1), respectively. For negative selection, normal human IgG (FujiFilm Wako, Osaka, Japan, #143-09501) immobilized beads were prepared and incubated with the library to remove non-specific Fc-reactive sequences.

SELEX Procedure

The selection was conducted in D-PBS-T buffer (D-PBS + 0.05% Tween 20). The ssDNA pool was denatured at 95 °C for 5 min and snap-cooled. After refolding, the library was incubated with target-immobilized beads on ice for 10–30 min. Unbound sequences were washed away, and bound sequences were eluted by heat (95°C for 10 min). On rounds 4–6, His-tagged FLT-1 was used to avoid tag-specific bias. In total, 7 rounds of selection were conducted (Supplementary Table S1). Each round included PCR amplification with Ds/PxTP and recovery of the ssDNA strand. Deep sequencing (Ion PGM, Thermo Fisher Scientific, MA, US.) was performed after the final round.

Sequence Analysis and Candidate Selection

Deep sequencing data were analyzed to identify dominant sequence clusters. Eleven major clusters were identified, from which 7 candidates were selected based on predicted secondary structures with stem-loop motifs (Supplementary Table S2).

ELOSA analysis

Based on deep sequencing analysis, several aptamer candidates were selected, and the binding activities of seven major sequences were evaluated using ELOSA (enzyme-linked oligonucleotide sorbent assay, summarized in Supplementary Fig. S1a). In this assay, 5 pmol of each biotinylated candidate sequence was immobilized on NeutrAvidin-coated plates (Pierce, Thermo Fisher Scientific, MA, USA) by incubation for 30 minutes at room temperature. His-tagged FLT-1 recombinant protein (Sino Biological, #10136-H08H, Ser27-Asn756-His, 400 nM), preincubated with 1 mg/mL salmon sperm DNA, was then added and incubated for 30 minutes at room temperature. After washing with 1x D-PBS-T, bound FLT-1 was detected using an HRP-conjugated anti-His antibody (R&D Systems, MN, USA), followed by color development with a substrate solution (R&D Systems, MN, USA). Absorbance at 450 nm (OD_{450}) was measured to assess binding activity. Candidate aptamers (e.g., flt-05, -07, -11) were tested by ELOSA using immobilized recombinant FLT-1, revealing strong binders (Supplementary Fig. S1b).

Secondary (Doped) SELEX and Optimization

Of the three high-affinity sequences identified in the ELOSA screen, we selected flt-07 for subsequent experiments because its predicted structure was the simplest, which facilitated chemical synthesis and downstream modifications. Based on its strong binding affinity, flt-07 was then subjected to sequence optimization using a doped library. Four rounds of secondary SELEX were conducted (Supplementary Table S3), and the optimized minimal sequence was selected based on secondary structure conservation. This sequence was further modified with a mini-hairpin at its 3′-end to generate TXB-0080.

TXB-0080 conjugated Sepharose preparation

TXB-0080 was conjugated to Sepharose beads using strain-promoted azide–alkyne cycloaddition (SPAAC) chemistry. First, 220 μM TXB-0080 containing an amino-modified linker was incubated with 1.5 mM DBCO–NHS (dibenzocyclooctyne–N-hydroxysuccinimidyl ester; Merck, MA, USA) in 0.1 M $NaHCO_3$ buffer (pH 8.7) at room temperature for 1 hour to obtain TXB-0080–DBCO.

For Sepharose modification, ω-aminohexyl–Sepharose™ 4B (Merck, MA, USA) was reacted with 100 mM NHS–PEG_4–N_3 (Tokyo Chemical Industry, Tokyo, Japan) in 0.1 M $NaHCO_3$ buffer (pH 8.7) for 2 hours at room temperature with gentle rotation. After washing three times with Milli-Q water, 250 nM TXB-0080–DBCO in Milli-Q water was incubated with the beads for 3 days at room temperature with rotation.

The reaction efficiency after TXB-0080–DBCO preparation and after Sepharose conjugation was confirmed by quantifying the remaining TXB-0080 (or TXB-0080–DBCO) in the supernatant using HPLC. HPLC analysis was performed on an XBridge C18 column (3.5 µm; Waters, MA, USA) with a mobile phase consisting of solvent A (95 mM triethylammonium acetate, 5% acetonitrile) and solvent B (acetonitrile). A linear gradient from 5% to 60% solvent B was applied at 0.75% per minute over 20 minutes using an Alliance 2695 HPLC system (Waters, MA, USA). The aptamer was detected by UV absorbance at 254 nm with a 2998 photodiode array detector (Waters, MA, USA). The conjugated beads were stored in 1x D-PBS containing 5 mM EDTA until use.

Quantification of TXB-0080 conjugated on Sepharose beads

To determine the amount of TXB-0080 conjugated to Sepharose beads, quantitative PCR (qPCR) analysis was performed using the beads as the template. TXB-0080–conjugated Sepharose beads were diluted with 25 mM HEPES–KOH buffer (pH 7.5; Nacalai Tesque, Kyoto, Japan). The diluted beads were mixed with 1× KOD SYBR qPCR Mix (TOYOBO, Osaka, Japan), 20 µM forward primer (5′-CTGACCCAGCGCCGCCGAAGG-3′), and 20 µM reverse primer (5′-GCGCTGACCCGTCCCCTCATCC-3′).

qPCR was carried out at 98 °C for 2 min, followed by 40 cycles of 98 °C for 10 s and 68 °C for 30 s. The amount of immobilized TXB-0080 per unit volume of beads was quantified based on a standard curve generated using free (unconjugated) TXB-0080 and the bead dilution factor.

Replicate 1:

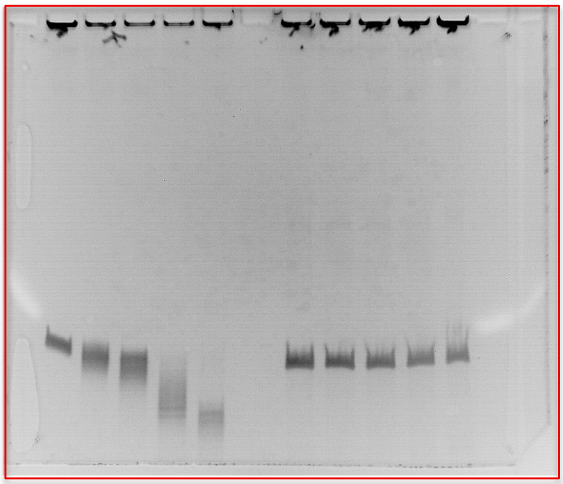

Replicate 2:

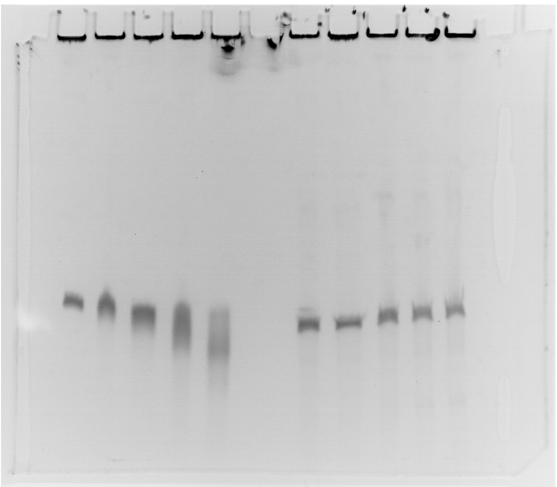

Replicate 3:

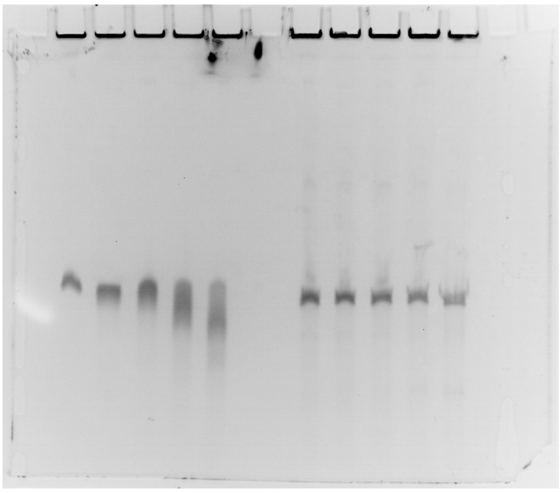

Full-length images for electrophoresis gels in Figure 2c.

The red boxes indicate the areas cropped for Figure 2c in the main manuscript.
